# *Cryptosporidium*-host interactions: What**’**s new?

**DOI:** 10.1016/j.crpvbd.2025.100285

**Published:** 2025-06-19

**Authors:** Dima Abdallah, Eric Viscogliosi, Gabriela Certad

**Affiliations:** a Université de Lille, CNRS, Inserm, CHU Lille, Institut Pasteur de Lille, U1019 - UMR 9017 - CIIL - Centre d’Infection et d’Immunité de Lille, 59000, Lille, France; b Délégation à la Recherche Clinique et à l’Innovation, Groupement des Hôpitaux de l’Institut Catholique de Lille, Lille Catholic University, 59000, Lille, France

**Keywords:** *Cryptosporidium-*host interactions, Immune evasion, Signalling pathways, Cryspoviruses, Cellular transformation, Microbiota, Viral co-infections

## Abstract

Species of *Cryptosporidium* are a leading cause of diarrhoeal disease worldwide, with severe outcomes in immunocompromised individuals and malnourished children. Despite the significant public health impact, no effective drug exists for these vulnerable populations. How *Cryptosporidium* spp. interact with the host remains incompletely understood. However, recent technological advances have begun to uncover novel mechanisms involved in parasite attachment, invasion, immune evasion through host pathway manipulation, potential host cell transformation, interactions with the gut microbiota, and modulation of viral co-infections. In this review, we synthesise these recent findings, offering an updated perspective on host-parasite dynamics and their implications for new therapeutic strategies.

## Highlights

• Recent findings reveal novel parasite proteins involved in host cell attachment and invasion.
• *Cryptosporidium* hijacks host signalling pathways to evade defenses and survive.
• *Cryptosporidium* harbours cryspoviruses that modulate host immunity and gene expression.
• Evidence suggests *Cryptosporidium* may contribute to colorectal cancer *via* epigenetic changes.
• Interactions with gut microbiota and co-infecting viruses influence *Cryptosporidium* infection.

## Graphical abstract

**Figure:**
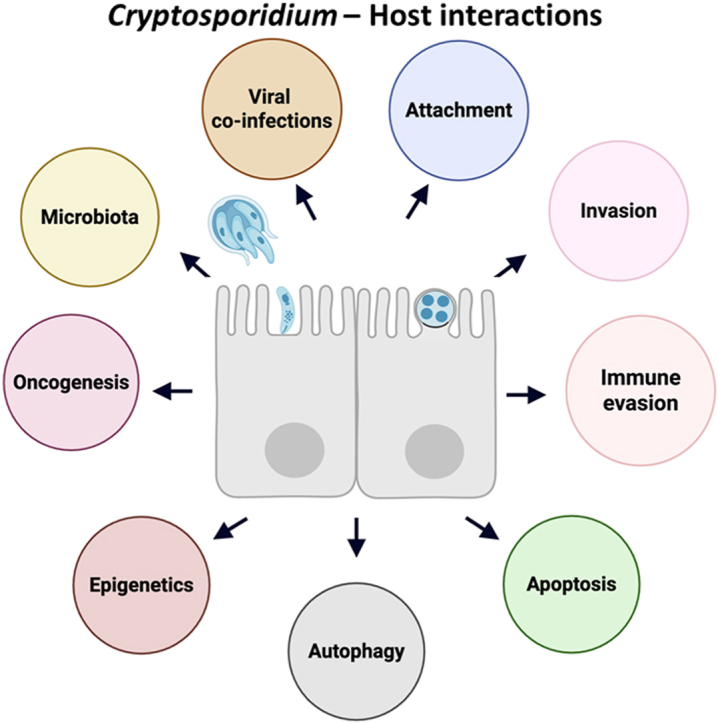

## 1 Introduction

*Cryptosporidium* spp., single-celled apicomplexan protozoan parasites, remain as a major public health concern, being one of the leading pathogen groups responsible for diarrhoeal diseases globally (Mohammed et al., 2017). According to the 2021 Global Burden of Diseases (GBD), *Cryptosporidium* spp. were the fourth leading cause of diarrhoeal mortality in children under five years of age (Kyu et al., 2025). Additionally, findings from GBD 2016 indicated that *Cryptosporidium*-induced diarrhoea in children from low-income settings has long-term health consequences, including impaired growth, particularly in weight, and an elevated risk of future infectious disease episodes (Khalil et al., 2018). In contrast, a recent Swedish study reported that children from high-income settings affected by *Cryptosporidium hominis* infection exhibited only mild long-term symptoms, such as fatigue and joint pain (Boks et al., 2025), suggesting that the long-term impact may vary according to socio-economic and environmental factors.

More than 40 species of *Cryptosporidium* have been documented, with the zoonotic *C. parvum* and the anthroponotic *C. hominis* being the species most commonly responsible for human infections. Transmission occurs primarily *via* the faecal-oral route, through ingestion of food or water contaminated with infective oocysts, or through direct contact with an infected host. These oocysts are highly stable in the environment and resistant to common water disinfection methods such as chlorination, making them difficult to eliminate from water facilities. Indeed, *Cryptosporidium* spp. are responsible for approximately half of all waterborne disease outbreaks in the USA (Ryan et al., 2021) and are also the leading cause of food- and waterborne outbreaks in Europe, with several large outbreaks reported in 2023 linked to contaminated tap water (EFSA and ECDC, 2024). While cryptosporidiosis typically manifests as a self-limiting disease in immunocompetent individuals, it can become severe, chronic, and even life-threatening in vulnerable populations, such as immunocompromised individuals, malnourished children, and those undergoing immunosuppressive therapies (Chalmers and Davies, 2010). Treatment options remain limited. Indeed, nitazoxanide, the only drug approved by the US Food and Drug Administration (FDA) for cryptosporidiosis, is effective in individuals with healthy immune systems but offers limited benefit for immunocompromised patients and young children (Khan and Witola, 2023).

The interactions between *Cryptosporidium* and host intestinal epithelial cells have long been incompletely understood due to the unique intracellular but extra-cytoplasmic location of the parasite, its capacity to evade immune defenses, and the historically limited number of identified virulence factors. These challenges complicate the understanding of parasite pathogenicity and hinder the development of effective interventions (Pinto and Vinayak, 2021). However, recent research has revealed novel insights into *Cryptosporidium*-host interplay, including processes of host-cell attachment and invasion, the subversion of host signalling pathways to evade immunity and survive intracellularly, the manipulation of apoptotic pathways, the triggering of cellular transformation, and the latest findings regarding its interplay with the gut microbiota, and modulation of viral co-infections. This review will focus on these recent discoveries, published primarily within the last two years, offering new insights into *Cryptosporidium* biology.

## 2 Unravelling *Cryptosporidium* adhesion

*Cryptosporidium* spp., unlike other apicomplexans, complete the entire life cycle within a single host. Infection begins with the ingestion of thick-walled oocysts, which undergo excystation in the gastrointestinal tract and release four motile sporozoites. These sporozoites possess an apical complex characteristic of the Apicomplexa, comprising secretory organelles such as micronemes, dense granules, and a single rhoptry (Tandel et al., 2019).

**Fig. 1:**
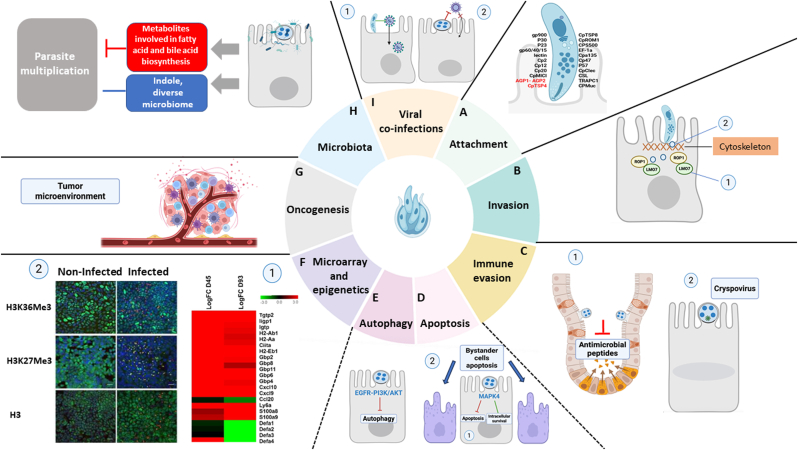
Recent advances contributing to the understanding of host-parasite dynamics. **A***Attachment*: Attachment to the host cell is facilitated by various molecules present on the sporozoite surface that are capable of binding to receptors on the host cell. Recently, two additional molecules (marked in red) have been identified: AGP1-AGP2 and CpTSP4. **B***Invasion*: (1) ROP1 is discharged and injected into the host cell during invasion, where it binds to the host actin cytoskeletal regulator LMO7; (2) Specialised organelles (blue circles) involved in host-parasite interface remodelling have been identified. These organelles release proteins that help in anchoring the parasite to its unique epicellular niche. **C***Immune evasion*: (1) To evade immune clearance, *Cryptosporidium* may suppress the expression of specific chemokines, including CCL20, β-defensin, and α-defensin, thereby impairing key components of the innate immune response, such as antimicrobial peptide activity; (2) Cryspoviruses appear to be key players in how the parasite infects and survives within the host (see Fig. 2 for more details). **D***Apoptosis* (although this process contributes to immune evasion as indicated by the dotted line, it warrants a separate description): (1) *Cryptosporidium* hijacks the host atypical Mitogen-activated protein kinase 4 (MAPK4) pathway to limit apoptosis and promote intracellular survival; (2) *Cryptosporidium* induces significant epithelial cell loss through apoptosis in bystander cells rather than in infected ones. **E***Autophagy* (although this process also plays a role in immune evasion, as shown by the dotted line, it deserves to be described separately): *Cryptosporidium* subverts autophagy by activating the EGFR-PI3K/AKT signalling cascade in order to promote intracellular survival. **F***Microarray and epigenetics*: (1) *Cryptosporidium* infection triggers profound changes in host gene expression, particularly in pro-inflammatory and pro-tumorigenic cytokines (figure modified from Sawant et al., 2021); (2) Immunofluorescence analysis of histone methylation events during *C. parvum* infection *in vitro* at 55 hours post-infection showed how the parasite has an effect on host histone lysine methylation leading to a considerable decrease in host H3K36me3 and H3K27me3 levels (photomicrograph modified from Sawant et al., 2022). **G***Oncogenesis:* Persistent *Cryptosporidium parvum* infection leads to the development of a tumor microenvironment in an experimental mouse model. **H***Microbiota*: The relationship between *Cryptosporidium* and the gut microbiota remains unclear, as studies have reported conflicting findings. While some suggest that the microbiota inhibits parasite replication, others indicate it may actually support the infection. **I***Viral co-infections*: (1) Sporozoite presence enhances bovine coronavirus entry into host epithelial cells; (2) Prior *C. parvum* or *C. hominis* infection inhibits subsequent rotavirus infection. This figure was created with BioRender.com.

Attachment to the host cell is facilitated by various molecules present on the sporozoite surface, which are capable of binding to receptors on the host cell (Fig. 1A). Key players include parasite mucin-like glycoproteins (e.g. GP40/15 and GP900) and several lectins that bind host cell carbohydrates (Lendner and Daugschies, 2014). Recently, an apical secretory glycoprotein complex, comprising AGP1-AGP2 heterodimers on *C. parvum* surface, was identified using the previously characterised monoclonal antibody MAb 1A5, which specifically stains the apical region of both sporozoites and merozoites. This complex was associated with additional orthologs found in the genome of closely related species. Furthermore, antibody neutralization of this complex was able to inhibit attachment and reduce infection rates (Akey et al., 2023). This finding expands the currently known repertoire of adhesive glycoproteins of *C. parvum*. Another significant breakthrough involves the discovery of a novel micronemal protein in *C. parvum*, Thrombospondin-repeat domain-containing protein-4 (CpTSP4). Immunostaining and enzyme-linked assays showed that this protein is pre-stored in micronemes and released during excystation, gliding and invasion. Notably, recombinant CpTSP4 displays nanomolar affinity to host cells and harbours a heparin-binding motif, implying heparin as one of its potential attachment ligands (Wang et al., 2024). Apart from CpTSP4, only a few other micronemal proteins have been experimentally verified by immunofluorescence assay (IFA) and/or immunoelectron microscopy (IEM) to localise to the anterior region of sporozoites, including GP900 (Lendner and Daugschies, 2014), TRAP-C1 (John et al., 2023), CpTSP8 (Putignani et al., 2008), and CpROM1 (Li et al., 2016).

## 3 A missing link in *Cryptosporidium* invasion

The exact mechanism by which *Cryptosporidium* spp. invade host cells is not completely understood. One key feature of *Cryptosporidium*-host interaction during invasion is host actin polymerization at the infection site. While actin polymerization depends on host cell's Arp2/3 complex, the parasite factors, as well as the transmitters and receptors that trigger this event, remain unknown (Li et al., 2016). Recent work using video microscopy combined with genetically encoded reporters to monitor both the parasite and host cell interaction during invasion, identified an effector rhoptry protein called ROP1 through transcriptional profiling (Fig. 1B-1). This protein is discharged and injected into the host cell during invasion, where it binds to the host actin cytoskeletal regulator LMO7. Interestingly, knocking out ROP1 significantly reduced parasite burden, while knocking out host LMO7 resulted in the loss of ROP1 accumulation in the terminal actin web and a significant increase in parasite infection. It is thus hypothesised that *C. parvum* injects ROP1 to interact with LMO7, hijacking host actin dynamics to facilitate invasion (Guérin et al., 2021).

**Fig. 2:**
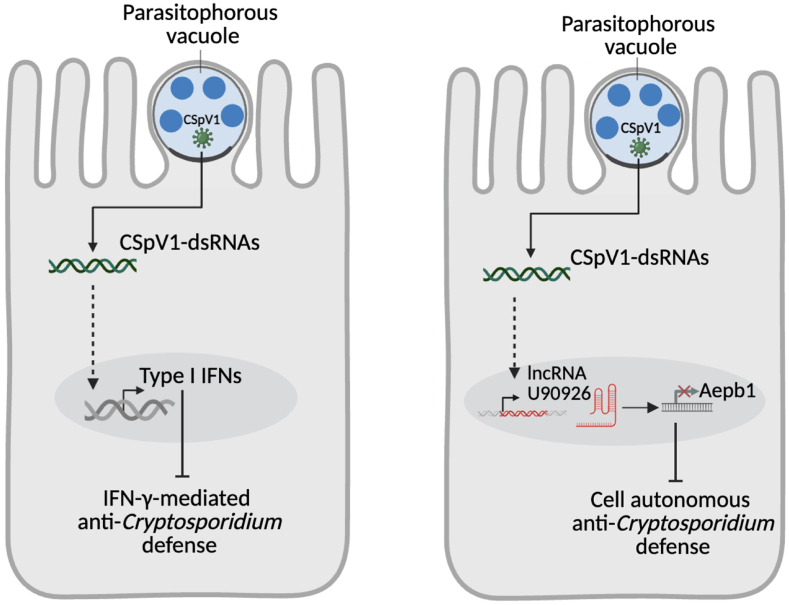
*Cryptosporidium parvum* employs distinct CSpV1-mediated strategies to evade intestinal epithelial cell antiparasitic defenses. In the left schematic, *C. parvum* delivers CSpV1-dsRNAs into the cytoplasm of the host intestinal epithelial cell, triggering the type I IFN signalling pathway, which in turn suppresses IFN-γ-mediated epithelial anti-*Cryptosporidium* responses. In the right schematic, delivery of CSpV1-dsRNAs leads to the upregulation of the long non-coding RNA U90926, which epigenetically represses Aepb1 expression, thereby attenuating cell-autonomous anti-*Cryptosporidium* defenses. This figure was created with BioRender.com.

More recently, a whole-cell spatial proteome of *C*. *parvum* sporozoite was analysed, and previously unidentified organelles contributing to the parasite's ability to remodel the host cell interface were discovered. These organelles release proteins that assemble into structures anchoring the parasite to its unique epicellular niche (Fig. 1, Fig. 2) (Guérin et al., 2023).

## 4 The art of survival: How *Cryptosporidium* evades and persists

After attaching to and subsequently invading intestinal epithelial cells, the sporozoite resides in a specialised epicellular compartment known as the parasitophorous vacuole (PV), which lies beneath the plasma membrane but separates the parasite from the host cytoplasm. Within this vacuole, the parasite undergoes both asexual and sexual stages, eventually leading to the shedding of sporulated oocysts in the faeces of the infected host (Tandel et al., 2019).

Epithelial cells activate multiple defense mechanisms in response to *Cryptosporidium* infection. They secrete cytokines and chemokines to recruit immune cells to the infection site, and release antimicrobial peptides capable of targeting and killing the parasite free stages (Laurent and Lacroix-Lamandé, 2017). *Cryptosporidium* spp. have evolved sophisticated strategies to evade these host immune defenses and ensure intracellular survival throughout the life cycle. A key feature is precisely this unique intracellular yet extra-cytoplasmic location within the PV. This positioning limits direct exposure of parasite antigens to immune cells, protects the parasite from phagocytosis, and prevents targeting by guanylate-binding proteins, which typically rupture the vacuoles of fully intracytoplasmic parasites (Mead, 2023). Additionally, *C. parvum* has evolved tactics to undermine certain components of the innate immune system such as the antimicrobial peptides. For instance, to avoid immune clearance, *C. parvum* may downregulate the expression of certain chemokines such as CCL20, β-defensin or α-defensin (Sawant et al., 2021) (Fig. 1C-1). *Cryptosporidium parvum* lacks the tricarboxylic acid cycle (TCA), the electron transport chain, and a functional ATP synthase, which makes the parasite dependent on glycolysis for energy production (Mogi and Kita, 2010). Recent research has revealed that *C. parvum* also ensures its intracellular survival by exploiting host cell metabolic pathways. Indeed, Xu et al. (2024) demonstrated that *C. parvum* can directly import phosphorylated hexoses from the host cell *via* two glucose transporters, CpGT1 and CpGT2, located at the parasite-host interface, thereby bypassing the need for its own hexokinase. Additionally, the parasite can access phosphorylated glucose from its internal amylopectin stores through a glycogen phosphorylase-dependent mechanism, which has been shown to be critical for its replication and survival (Xu et al., 2024).

Various other mechanisms contribute to immune evasion and intracellular survival. For example, *Cryptosporidium* spp. have evolved mechanisms to regulate apoptosis and epithelial cell turnover in a stage-specific manner, promoting parasites’ own growth and ensuring adequate time to complete the life cycle (Certad, 2022). In particular, numerous studies have highlighted the critical role of NF-κB in the host cell response to the infection. This transcription factor, which can be activated through Toll-like receptors, is involved in both pro-apoptotic and anti-apoptotic signalling pathways (Certad, 2022). Parasite-driven production of other host anti-apoptotic factors such as BCL-2, survivin, and osteoprotegerin has also been described (Mead, 2023). A recent study has uncovered a novel layer of this interaction, revealing that *C. parvum* hijacks the host atypical Mitogen-activated protein kinase 4 (MAPK4) pathway to limit apoptosis and promote intracellular survival (Fig. 1D-1). Indeed, knocking out MAPK4 from HCT-8 colon adenocarcinoma cells significantly reduced infection, correlating with increased caspase-dependent apoptotic cell death induced by the parasite. In addition to its anti-apoptotic role, MAPK4 was also implicated in facilitating parasite invasion and asexual reproduction, indicating a broader role in the infection process. Nevertheless, the precise molecular mechanisms through which *C. parvum* interacts with MAPK4 have yet to be fully defined (Watanabe et al., 2023). Consistently, other MAPK have been described as modulated by the infection: a marked downregulation of p38/MAPK, MAP kinase-activated protein kinase 2 (Mk2), and Mk3 gene expression was observed in an *in vitro* model of cryptosporidiosis. The suppression of MAPK signalling was associated with a weakened intestinal epithelial defense against *C. parvum* infection (He et al., 2021).

Further expanding on the parasite relationship with apoptosis, a prominent study investigating intestinal epithelial architecture during infection found that *C. parvum* induces significant epithelial cell loss in murine intestines through apoptosis in bystander cells rather than in infected ones (Fig. 1, Fig. 2) (Wallbank et al., 2024). Indeed, complementary *in vitro* experiments showed that infected HCT-8 cells treated with apoptosis-inducing agents exhibited significantly fewer apoptotic markers compared to uninfected bystander cells, suggesting that *C. parvum* confers resistance to chemically induced apoptosis (Wallbank et al., 2024).

Beyond apoptosis, it was recently discovered that *C. parvum* also interferes with autophagy, a lysosomal-dependent degradation process essential for degrading intracellular pathogens (Pavlik et al., 2024). Yang et al. (2023) demonstrated that *C. parvum* subverts autophagy by activating the EGFR-PI3K/AKT signalling cascade in HCT-8 cells, which is known to negatively regulate autophagic processes (Fig. 1E). By hijacking this pathway, *C. parvum* effectively inhibits autophagy and promotes its intracellular survival. On the other hand, inhibition of either EGFR or AKT decreased the number of intracellular parasites. Further studies are needed to validate these findings in non-transformed intestinal epithelial cell models, as HCT-8 cells are already transformed.

## 5 Cryspoviruses: Mysterious *Cryptosporidium* roommates

Extrachromosomal double-stranded RNA (dsRNA) viruses are harboured by several protozoan parasites such as *Trichomonas*, *Giardia*, *Leishmania*, and *Cryptosporidium*. The *Cryptosporidium parvum virus 1* (CSpV1), which belongs to the genus *Cryspovirus* and family *Partitiviridae*, was first discovered in the cytoplasm of *C. parvum* sporozoites in 1997. CSpV1 is a non-enveloped virus with a bi-segmented genome, consisting of dsRNA1 and dsRNA2, with each segment encoding a distinct protein (Khramtsov et al., 1997). The impact of such viruses on host-parasite interactions is exemplified by the *Leishmania RNA virus* (LRV), which is strongly associated with the development of mucocutaneous leishmaniasis, the severe form of the disease. LRV subverts host immune responses *via* Toll-like receptor 3 (TLR3)-mediated activation of type I interferon (IFN) production, leading to chronic inflammation and tissue destruction (De Carvalho et al., 2019).

Although CSpV1 was identified decades ago, its overall impact on *C. parvum* virulence had not been characterised until recently. Studies now report that during infection, *C. parvum* delivers CSpV1 dsRNAs into the cytoplasm of host intestinal epithelial cells (Fig. 1, Fig. 2 and Fig. 2). These dsRNAs activate the type I IFN signalling pathway, which, rather than enhancing protection, suppresses IFN-γ-mediated intestinal epithelial cell antiparasitic immunity. This unveils a novel immune evasion strategy in which the parasite exploits host signalling pathways to evade antiparasitic defenses (Deng et al., 2023).

Adding another layer to immune evasion, *C. parv*um utilises CSpV1 to upregulate a long noncoding RNA (lncRNA) named U90926 in infected intestinal epithelial cells (Fig. 1, Fig. 2 and Fig. 2). Silencing U90926 significantly reduced parasite burden, while its overexpression exacerbated infection severity. This lncRNA was shown to suppress transcription of Aebp1, a gene encoding a protein critical for epithelial cell-autonomous antiparasitic defense, through epigenetic regulation. Taken together, these findings underscore how *C. parvum* leverages CSpV1 to manipulate host lncRNA pathways, suppress immune defenses, and ensure intracellular survival (Graham et al., 2023). While cryspoviruses appear to be more friends than foes to *Cryptosporidium*, their exact role is still under investigation. They may yet prove to be key players in how the parasite infects and survives within the host.

## 6 *Cryptosporidium,* a pirate of cellular pathways

*Cryptosporidium* infection is not a passive event; rather, as already discussed, the parasite actively reprograms key host cellular processes, including signalling pathways, structural components, and immune responses, to promote its replication and persistence while simultaneously weakening host defenses. Within this context, an expanding body of evidence suggests that *C. parvum* may act as a potential oncogenic agent. In fact, epidemiological studies have reported significantly higher infection rates among newly diagnosed colorectal cancer patients compared to control groups, with infection rates of 21% in Lebanon (Osman et al., 2017), 17.24% in China (Zhang et al., 2020), and 32.5% in Egypt (Abd El-Latif et al., 2023). Experimental studies further support a potential oncogenic role for *C. parvum*. In murine models, the parasite has been shown to induce gastrointestinal adenocarcinomas as early as 45 days post-infection (Certad et al., 2007). Complementary *ex vivo* experiments using cultured murine colonic explants have also demonstrated the development of neoplastic lesions within 27 days following infection (Baydoun et al., 2017), reinforcing the hypothesis that *C. parvum* contributes directly to tumorigenesis. Notably, infectious agents are implicated in approximately 20% of the global cancer burden, and projections estimate that infections could account for the majority of cancer cases worldwide by 2050 (De Martel et al., 2020).

At the molecular level, *C. parvum* infection triggers profound changes in host gene expression, particularly in intestinal epithelial cells. Transcriptomic analyses of infected cells from severe combined immunodeficient (SCID) mice revealed modulation of pro-inflammatory and pro-tumorigenic cytokines (Fig. 1F-1). These cytokines not only suppress apoptosis and facilitate immune evasion but also sustain chronic inflammation and promote an immunosuppressive microenvironment (Fig. 1G), conditions that are conducive to tumor development (Sawant et al., 2021).

More recently, studies have shed light on epigenetic modifications induced by *C. parvum* infection. Notably, significant losses of histone H3K36me3 and H3K27me3 methylation were observed both *in vitro* and *in vivo* (Fig. 1, Fig. 2). These histone marks are critical for the regulation of gene expression and genomic stability: H3K27me3, regulated by the Polycomb repressive complex 2 (PRC2) and its catalytic subunit EZH2, is frequently deregulated in cancer, while H3K36me3 is involved in transcriptional fidelity, mRNA splicing, and DNA repair, with its disruption linked to various human malignancies (Sawant et al., 2022).

Taken together, these findings confirm that *Cryptosporidium*'s ability to hijack host signalling and epigenetic regulatory pathways likely contributes to host cell transformation as already suggested by others (Hussain et al., 2025). However, further research is urgently needed to fully elucidate the underlying mechanisms and to establish a definitive causal relationship between *Cryptosporidium* infection and cancer development.

## 7 *Cryptosporidium* and microbiota: Friends or foes?

The relationship between *Cryptosporidium* spp. and the gut microbiota is not clear, with studies reporting conflicting results (Fig. 1H). Some studies suggest that the microbiota inhibits parasite multiplication, while others indicate that microbiota may be beneficial for the infection. Concerning the inhibitory role of microbiota, it has been reported that certain gut microbiota-derived metabolites, such as indoles, can inhibit *C. parvum* growth both *in vitro* and *in vivo*. This inhibitory effect occurs through disruption of the host cell mitochondrial respiratory chain, leading to reduced ATP production, an essential energy source for the parasite. Indoles have also been shown to reduce the membrane potential of the *C. parvum* mitosome, a remnant mitochondrial organelle involved in iron-sulfur cluster assembly and ubiquinone biosynthesis (Funkhouser-Jones et al., 2023).

Conversely, research by Wang et al. (2023) demonstrated that infection of mice with *C. muris*, a species that primarily infects rodents, resulted in an increased abundance of bacterial taxa like *Lachnospiraceae* and *Prevotella*, which are associated with the production of metabolites involved in unsaturated fatty acid and primary bile acid biosynthesis. Notably, metabolites related to bile acid pathways were significantly elevated at the peak of parasite growth and oocyst shedding, suggesting a supportive role in parasite development and infection progression. However, it is important to note that *C. muris* is a parasite of the stomach; thus, the comparison with intestinal forms of cryptosporidiosis caused by *C. parvum* or *C. hominis* is limited due to differences in infection sites and host-microbiota dynamics. Nevertheless, these findings highlight the potential for species- and site-specific microbiota changes to influence parasite development. Further studies infecting relevant intestinal models with *C. parvum* or *C. hominis* are warranted to determine whether similar microbiota-driven mechanisms operate in human cryptosporidiosis.

On the other hand, different studies have analysed the association between variations in microbiome composition and the severity of infection. A systematic literature review revealed that a diverse microbiome prior to infection, whether naturally present or experimentally induced, is associated with reduced infection severity and lower oocyst output (Hurle et al., 2023). Similarly, Morita et al. (2024) found that neonatal calves infected with *C. parvum* had higher gut microbiota α-diversity in non-diarrhoeal faecal samples compared to diarrhoeal ones, with *Megasphaera* spp. being more abundant in healthy samples. This observation is consistent with a previous study among Bangladeshi infants infected with *Cryptosporidium* spp., which found that *Megasphaera* spp. was significantly less abundant in the faecal samples of infants with diarrhoea compared to those who remained asymptomatic (Carey et al., 2021).

Furthermore, recent studies indicate that interventions such as dietary modifications, probiotics, and even faecal microbiota transplantation (FMT) may help restore microbial diversity and reduce the severity of cryptosporidiosis, especially in vulnerable populations where conventional treatments often fail (Ali et al., 2025). It is important to note that while these interventions seem promising, their efficacy can vary based on factors such as the specific probiotic strains used and the timing of administration. Some studies have reported mixed outcomes, indicating the necessity for more targeted research to optimise these therapeutic strategies.

Overall, further research is needed to comprehensively understand how variations in microbiome composition influence infection severity, identify specific genera that may offer protection, and determine those that may worsen disease pathology.

## 8 *Cryptosporidium* and viral co-infections

Beyond its interactions with the gut microbiota, *Cryptosporidium* spp. also appear to modulate viral co-infections within the host. For instance, simultaneous co-inoculation of HCT-8 cells with *C. parvum* and bovine coronavirus increased viral entry into host cells when *C. parvum* sporozoites were also present (Fig. 1I-1), although this did not result in higher viral copy numbers after 24 hours (Shakya et al., 2022). Similarly, studies in swine have reported a higher prevalence of Hepatitis E Virus (HEV) in animals infected with *Cryptosporidium* spp. compared to non-infected counterparts. This pattern was not observed with extracellular enteroparasites, suggesting a potential facilitative role of *Cryptosporidium* spp. in HEV infection (Rivero-Juárez et al., 2022). Conversely, *Cryptosporidium* spp. may also exert antiviral effects. *In vitro* studies using stem cell-derived human intestinal epithelium showed that prior infection with *C. parvum* or *C. hominis* inhibited subsequent rotavirus infection (Fig. 1, Fig. 2), likely through the induction of a type III interferon response (Greigert et al., 2024). These findings highlight the complex and context-dependent role of *Cryptosporidium* spp. in shaping intestinal viral ecology, though further research is needed to fully elucidate these interactions and their impact on disease severity.

## 9 Conclusions

Recent advances in our understanding of *Cryptosporidium-*host interactions are reshaping the field of cryptosporidiosis. From unique mechanisms of parasite attachment and invasion to sophisticated immune evasion strategies, and unexpected roles for *Cryptosporidium*-harboured viruses, microbiota-derived metabolites, and modulation of viral co-infections, a more complex picture of infection is emerging. These discoveries not only fill longstanding gaps but also reveal how *Cryptosporidium* spp. manipulate host cellular pathways to establish chronic infection, and potentially contribute to gastrointestinal tumorigenesis. The growing recognition of the bidirectional crosstalk between parasite and the gut microbiota opens up new therapeutic possibilities, including microbiota-targeted interventions. Meanwhile, the identification of cryspovirus-driven immune modulation highlights promising molecular targets for drug development. Despite these advances, many aspects of host-parasite interactions remain to be explored. The full repertoire of *Cryptosporidium* effectors, the identity of host receptors, and the precise mechanisms of invasion, immune evasion and host cell transformation remain elusive. A major challenge is that most *in vitro* studies rely on transformed cell lines like HCT-8, which cannot support the complete life cycle of the parasite, further limiting progress in the field. Bridging these knowledge gaps will require more physiologically relevant models that better replicate the complexity of the gut, along with the application of genetic tools that are already available today for manipulating the parasite. Such approaches will be helpful in advancing our understanding of *Cryptosporidium* interactions with intestinal epithelial cells. Altogether, the recent breakthroughs covered in the review offer not just deeper insight into *Cryptosporidium* biology but a clearer path toward therapeutic innovation, especially for the immunocompromised individuals and young malnourished children most affected by this persistent and challenging pathogen.

## CRediT authorship contribution statement

**Dima Abdallah:** Formal analysis, Writing – review & editing. **Eric Viscogliosi:** Writing – review & editing. **Gabriela Certad:** Conceptualization, Data curation, Visualization, Writing – original draft, Writing – review & editing.

## Ethical approval

Not applicable.

## Funding

This research was supported by the Centre National de la Recherche Scientifique (CNRS), the Institut National de la Santé et de la Recherche Médicale (INSERM), the Institute Pasteur de Lille, the Université de Lille, the Groupement des Hôpitaux de l’Institut Catholique de Lille, the Université Catholique de Lille, and the Centre Hospitalier Régional Universitaire de Lille. D. A. was supported by a PhD fellowship from the 10.13039/100015872Université de Lille.

## Declaration of competing interests

The authors declare that they have no known competing financial interests or personal relationships that could have appeared to influence the work reported in this paper.
